# Sharpen data-driven prediction rules of individual large earthquakes with aid of Fourier and Gauss

**DOI:** 10.1038/s41598-023-43181-z

**Published:** 2023-09-25

**Authors:** In Ho Cho

**Affiliations:** CCEE Department, Iowa State University, Ames, IA 50011 USA

**Keywords:** Natural hazards, Computational science

## Abstract

Predicting individual large earthquakes (EQs)’ locations, magnitudes, and timing remains unreachable. The author’s prior study shows that individual large EQs have unique signatures obtained from multi-layered data transformations. Via spatio-temporal convolutions, decades-long EQ catalog data are transformed into pseudo-physics quantities (e.g., energy, power, vorticity, and Laplacian), which turn into surface-like information via Gauss curvatures. Using these new features, a rule-learning machine learning approach unravels promising prediction rules. This paper suggests further data transformation via Fourier transformation (FT). Results show that FT-based new feature can help sharpen the prediction rules. Feasibility tests of large EQs ($M\ge$ 6.5) over the past 40 years in the western U.S. show promise, shedding light on data-driven prediction of individual large EQs. The handshake among ML methods, Fourier, and Gauss may help answer the long-standing enigma of seismogenesis.

## Introduction

Large earthquakes (EQs) remain one of the most difficult physics phenomena, attracting the newest technologies. Recently, researchers actively leverage machine learning (ML) methods^1,2^. For instance^3^, adopted deep neural networks (DNNs) to forecast aftershock locations without relying upon fault orientation. Their DNNs take co-seismically generated static elastic tensor’s change as input and produce binary prediction of whether each refined grid cubic cell (5 km each dimension) will contain aftershocks or not^4^. Reconstructed the time series data in EQ catalog of Southern California to a sequence of two-dimensional (2D) images, and they combined an autoencoder and temporal convolutional neural networks to the new data to predict the probability of extreme events. Reference^5^ adopts the long short-term memory networks to learn EQ’s spatio-temporal relationship. At the same time, deep learning helps to engender denser and deeper data sets of EQs^6,7^, which can enable unsupervised deep learning-driven exploration and discovery of hitherto unseen behaviors and patterns of EQ^8^. By regarding EQs as spatio-temporal point process, a combination of reinforcement learning and neural networks is used^9^ for enabling data-driven fitting and learning of heterogeneous Gaussian diffusion kernels to improve predictions of the point processes. Still, these ML-based approaches are at the burgeoning phase and require systematic validation and comparison against existing approaches. Reference^10^ conducted a comprehensive comparative study on neural networks-based EQ forecasting and prediction methods of the past three decades and found that these new methods call for broader and systematic validations since simple empirical methods may exhibit equivalent or even better performance.

Reference^11^ gives a comprehensive overview of recent physics-based EQ forecasting methods, for which a well-established performance evaluation framework is available by community^12,13^. Diverse ML methods play an important role in understanding EQs’ complex behaviors and patterns, e.g., Refs.^14–16^.

Despite the notable advances empowered by ML-driven approaches, the long-sought capability of predicting “individual” large EQs’ locations and magnitudes within a short time frame (days or weeks ahead) remains unreachable. This study seeks to add a new dimension to this daunting question. The author’s prior study^17^ shows that, after multi-layered data transformations, individual large EQs appear to have unique signatures that can be represented by new high-dimensional features. In particular, the observed EQ catalog data are transformed via spatio-temporal convolution, and then further transformed into a number of pseudo physics quantities (i.e., energy, power, vorticity, and Laplacian). They later turn into smooth surface-like information via Gauss curvatures, giving rise to new high-dimensional features. The new features of pseudo physics quantities are used to build a customized prediction model by the Bayesian evolutionary algorithm in conjunction with flexible base functions. Validations with the past 40-year EQs catalog data of the western U.S. region show that the Gauss curvature-based coordinates appear to hold uniqueness for individual large EQs ($M_w \ge 7.0$) demonstrating a promising reproduction of individual large EQs’ locations and magnitudes 30 days before the event.

Here, this study expanded the study region from (longitude, latitude, depth) = $[-130^{\circ }, -110^{\circ }]\cup [30^{\circ }, 45^{\circ }]\cup [-5\text { km}, 20\text { km}]$ to $[-132.5^{\circ }, -110^{\circ }]\cup [30^{\circ }, 52.5^{\circ }]\cup [-5\text { km}, 20\text { km}]$ and the magnitude range from $M_w \ge 7.0$ to $M_w \ge 6.5$. As a result, the total number of target large EQs within the 40 years (1980 through 2019) increases from 8 to 17. Importantly, this study adds further sophistication to previous multi-layered data transformations via Fourier transformation of the Gauss curvature-based features.

The key equations and formulas of the proposed approach are summarized in Table S1 of Supplementary Information.

**Figure 1:**
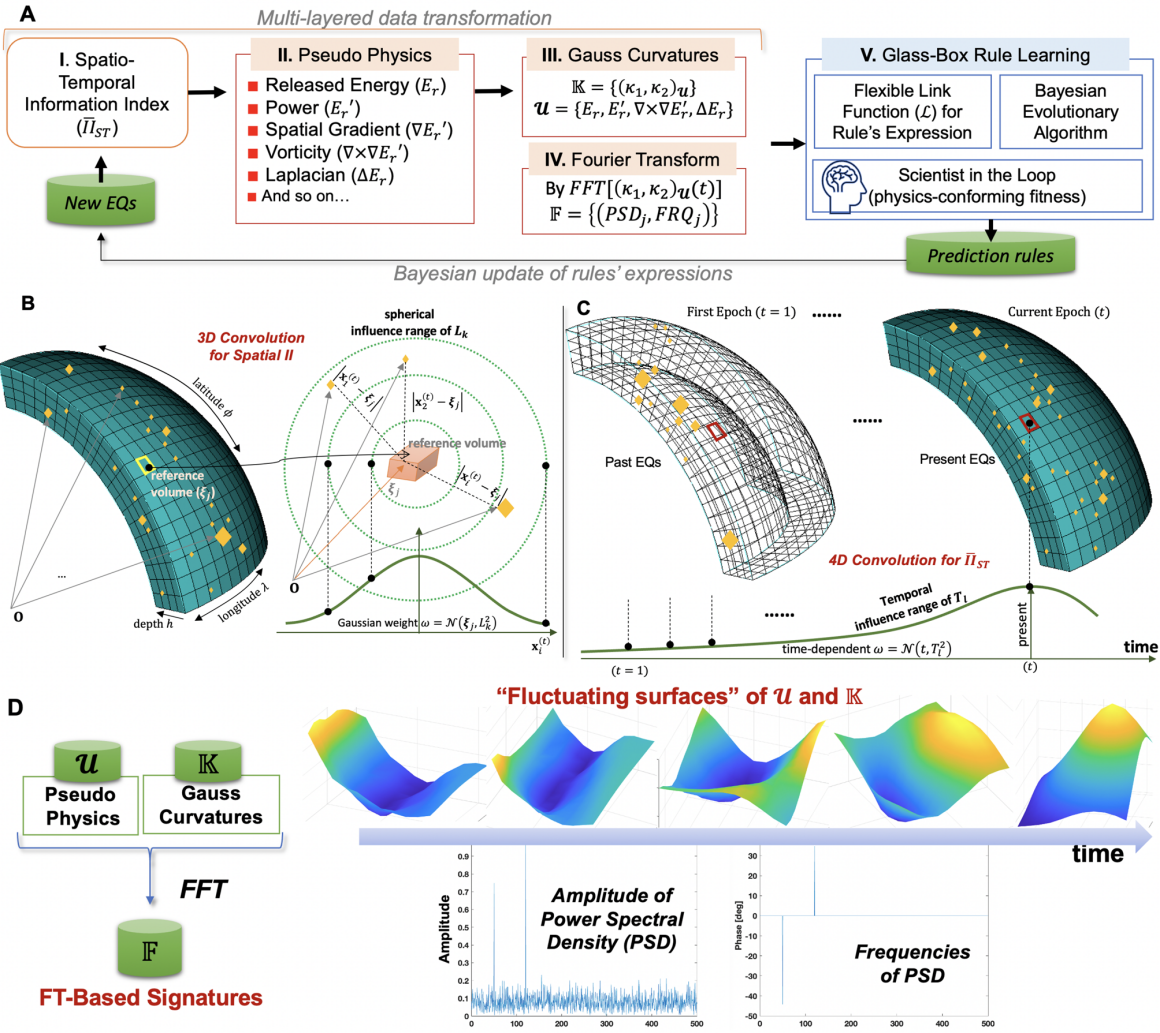
Overall architecture and conceptual illustrations of central steps of the proposed approach: (**A**) Multi-layered data transformation from raw EQs catalog data to new features in terms of pseudo physics quantities, Gauss curvatures, and Fourier bases (I–IV). Transparent rule-learning machine learning (ML) method, denoted “glass-box” ML (V), to unravel prediction rules’ expressions of individual large EQs; (**B**) 3D convolution for generating spatial information index; (**C**) Temporal convolution for spatio-temporal information index; (**D**) Fast Fourier transform (FT) to generate FT-based new feature that can quantify time-varying information about “fluctuating surfaces” of the pseudo physics and Gauss curvature-based features.

## Results

### Multi-layered data transformation

The critical novelty of the proposed approach lies in the multi-layered data transformation to generate physics-infused ML-friendly new features. The overall architecture of data transformation is summarized in Fig. 1

The first data transformation (Figs. 1B,C) converts the raw EQ catalog data (USGS^18^) into new scalar features, denoted as spatio-temporal information index (${\overline{II}}_{ST}$). The reference volume is defined as a discretized volume of the Earth lithosphere with increment of (longitude, latitude, depth), $(\Delta \lambda , \Delta \phi , \Delta h)=(0.1^{\circ }, 0.1^{\circ }, 5\text { km})$. For the convolution process, the geodetic coordinates, $(\lambda , \phi , -h)_{i}^{(t)}$ are transformed into the earth-centered rectilinear coordinate $(x, y, z)_{i}^{(t)}$^17^. Each observed EQ’s moment magnitude $M_{i}^{(t)}\in {\mathbb {R}}[0,10)$ are assumed to reside at ${{\textbf {x}}}_i^{(t)} = (x, y, z)_i^{(t)}$, following the “point source” concept. Physically, ${\overline{II}}_{ST}(\varvec{\xi }_{j})^{(t)}$ quantifies the accumulated influences of the adjacent EQs close to the *j*th reference volume center and of the past EQs up to the present time (*t*). The details of the data transformation from raw EQ catalog data into spatio-temporal information index generation are presented in “Methods”.

The second data transformation is to convert the spatio-temporal IIs into the pseudo physics quantities. Amongst many physics quantities, the best-so-far set of pseudo physics quantities are identified as {released energy, power, vorticity, Laplacian}^17^. To be purely data-driven, no pre-defined statistical or empirical laws are used. Instead, a flexible function, called “link function (LF)”, is used to learn expressions of the pseudo physics quantities. The best-so-far form of the pseudo released energy $E_r^{(t)}(\varvec{\xi }_{j})$ is identified as

1 $$\begin{aligned} E_r^{(t)}(\varvec{\xi }_{j}) = \sum _{k=1}^{n_L=2} \sum _{l=1}^{n_T=2} {\mathcal {L}}^{(k,l)}({\overline{II}}_{ST}^{(t)}(\varvec{\xi }_{j}; L_k, T_l); ~\varvec{\uptheta }^{(k,l)}), \end{aligned}$$

where $\varvec{\uptheta }^{(k,l)}$ is the best-so-far free parameters of the associated LF ${\mathcal {L}}^{(k,l)}$. Here, ${\mathcal {L}}^{(k,l)}$ takes ${\overline{II}}_{ST}^{(t)}(\varvec{\xi }_{j}; L_k, T_l)$ as input and produces a smooth, nonlinear output.

$L_k (k=1, \ldots ,n_L)$ is the spatial influence range that means the spatial proximity-dependent importance in the spatial convolution process. Similarly, $T_l (l=1, \ldots ,n_T)$ is the temporal influence range meaning the temporal proximity-dependent importance in the temporal convolution process (see “Methods” for details).

Any mathematical form can be used as LF, and a simple yet general exponential form LF works well^17^, i.e., for the pseudo released energy ${\mathcal {L}}^{(k,l)}({\overline{II}}_{ST}^{(t)}(\varvec{\xi }_{j}; L_k, T_l)~\varvec{\uptheta }^{(k,l)})$=$\text {exp}\left( a^{(k,l)}{\overline{II}}_{ST}^{(t)}(\varvec{\xi }_{j}; L_k, T_l)^{b^{(k,l)}}\right) - 1$ where $\varvec{\uptheta }^{(k,l)} = \{a^{(k,l)}, b^{(k,l)}; k=1, \ldots ,n_L, l=1,\ldots ,n_T\}$. The pseudo “vorticity” $\varvec{\omega } = (\omega _\lambda , \omega _\phi , \omega _h)$ is generated by $\varvec{\omega } := \nabla _g \times \left( \nabla _g{ \frac{\partial E_r^{(t)}(\varvec{\xi }_{j})}{\partial {t}}} \right)$ where $\frac{\partial E_r^{(t)}(\varvec{\xi }_{j})}{\partial {t}}$ corresponds to the pseudo “power” and the pseudo “Laplacian” is calculated as $\nabla _g^2 E_r^{(t)}(\varvec{\xi }_{j}) = \frac{\partial ^2{E_r^{'}}}{\partial {\lambda ^2}}+ \frac{\partial ^2{E_r^{'}}}{\partial {\phi ^2}} + \frac{\partial ^2{E_r^{'}}}{\partial {h^2}},$ where $\nabla _{g}(.)$ means the spatial gradient with respect to the geodetic coordinate system (${\lambda , \phi , h}$). As shown in Ref.^17^, amongst many pseudo physics quantities and their combinations, ML selected out the four quantities—the released energy, power, the first vorticity term, and the first Laplacian term, (${E}_{r}^{(t)}, \frac{\partial E_r^{(t)}(\varvec{\xi }_{j})}{\partial {t}}, \omega _{\lambda }, \frac{\partial ^2{E_r^{'}}}{\partial {\lambda ^2}}$), at least for the western U.S. region. Again, this selection is purely data-driven since ML simply seeks to find the best combination that can outperform other cases without any prejudice. The third data transformation is to convert the pseudo physics quantities into Gauss curvatures. At each depth, the distributions of the pseudo physics quantities constitute smooth yet complex surfaces. To effectively inform ML with surface-like information, the next data transformation focuses on Gauss curvatures^19^—consisting of two principal curvatures $\kappa _1$ and $\kappa _2$ (detailed calculation procedures are presented in Ref.^17^). Using the Gauss curvatures near EQs, it is easy to quantify the distributions’ shapes of the pseudo physics quantities. In Ref.^17^, these Gauss curvature-based coordinates may serve as a unique signature of individual extreme EQs. The coordinate vector *K* consists of the principal Gauss curvatures $(\kappa _1, \kappa _2)$ of four pseudo physics quantities at time *t* at a reference volume $\varvec{\xi }_j$ as

2 $$\begin{aligned} K(t; \varvec{\xi }_j):=\left( (\kappa _1, \kappa _2)_E, (\kappa _1, \kappa _2)_P, (\kappa _1, \kappa _2)_V, (\kappa _1, \kappa _2)_L)\right) , \end{aligned}$$

where *E*, *P*, *V* and *L* stand for the pseudo released energy, the pseudo power, the pseudo vorticity’s first term, and the pseudo Laplacian’s first term, respectively, all being calculated at time *t* and the reference volume $\varvec{\xi }_j$.

**Table 1:** Algorithm—Fourier transform (FT)-based new feature generation.

**[Step-I] Time-history of gauss curvatures of pseudo physics quantities**	
**Loop: ** $\forall \xi _j \in \text {V}$	
$$\;\;\;\;\;\;{\textbf {Loop: }} t = 1,..., t_n$$	
$$\;\;\;\;\;\;\;\;\; \text {Calculate} {K}(t;\xi _j) = ((\kappa _1, \kappa _2)_E, (\kappa _1, \kappa _2)_P, (\kappa _1, \kappa _2)_L, (\kappa _1, \kappa _2)_V)_j^{(t)}$$	
$$\;\;\;\;\;\;{\textbf {End Loop}}$$	
$$\;\;\;\;\;\; {\mathbb {K}}(n;\xi _j)=\begin{bmatrix} ((\kappa _1, \kappa _2)_E, (\kappa _1, \kappa _2)_P, (\kappa _1, \kappa _2)_L, (\kappa _1, \kappa _2)_V)_j^{(1)} \\ \vdots \\ ((\kappa _1, \kappa _2)_E, (\kappa _1, \kappa _2)_P, (\kappa _1, \kappa _2)_L, (\kappa _1, \kappa _2)_V)_j^{(t_n)} \\ \end{bmatrix} \in {\mathbb {R}}^{n \times 8}$$	
**End Loop**	

**[Step-II] Column-wise Fast Fourier Transform (FFT)**	
**Loop: ** $(\forall \xi _j \in \text {V})$	
$$\;\;\;\;\;\;{\textbf {Loop: }} (i = 1,..., 8)$$	
$$\;\;\;\;\;\;\;\;\;\;\;\; ({{\textbf {p}}}^{(i)}_{PSD}, {{\textbf {f}}}^{(i)}) = \text {FFT}[{{\textbf {k}}}^{(i)}(n;\xi _j) = i_{th} \text { column of } {\mathbb {K}}(n;\xi _j) ]$$	
$$\;\;\;\;\;\;\;\;\;\;\;\; (\overline{{{\textbf {p}}}}^{(i)}_{PSD}, \overline{{{\textbf {f}}}}^{(i)}) = \text {Sorted } ({{\textbf {p}}}^{(i)}_{PSD}, {{\textbf {f}}}^{(i)}) \text { by PSD in descending order}$$	
$$\;\;\;\;\;\;\;\;\;\;\;\; (\overline{{{\textbf {p}}}}^{(i)}_{top}, \overline{{{\textbf {f}}}}^{(i)}_{top}) = \text {Top 10 largest } (\overline{{{\textbf {p}}}}^{(i)}_{PSD}, \overline{{{\textbf {f}}}}^{(i)}) \text { by PSD}$$	
$$\;\;\;\;\;\;{\textbf {End Loop}}$$	
$$\;\;\;\;\;\; {\mathbb {F}}(n;\xi _j)=\begin{bmatrix} \overline{{{\textbf {p}}}}^{(1)}_{top}&\overline{{{\textbf {f}}}}^{(1)}_{top}&\dots \overline{{{\textbf {p}}}}^{(8)}_{top}&\overline{{{\textbf {f}}}}^{(8)}_{top} \end{bmatrix} \in {\mathbb {R}}^{10 \times 16}$$	
**End Loop**	

The fourth data transformation is to convert the time histories of Gauss curvatures into Fourier transform (FT)-based features (Fig. 1D). The inclusion of the FT-based new features has two reasons. First, FT-based features can easily convey temporal information of other features in terms of amplitudes and frequencies. The second reason is to leverage the strength of the Fourier series in representing general, complex functions. As shall be demonstrated in this paper, the inclusion of trigonometric functions (inspired by the Fourier series) in the prediction rules appears to improve and sharpen the prediction accuracy substantially.

Table 1 summarizes the key procedures of FT-based new feature generation using the time history of Gauss curvatures of pseudo physics quantities. The set of Gauss curvature-based coordinates at $\varvec{\xi }_j$ up to the present time $t_n = n\times \Delta t$ is given by

3 $$\begin{aligned} {\mathbb {K}}(n;\varvec{\xi }_j):=\{{K}(t;\xi _j) \in {\mathbb {R}}^8| \; t=1,\ldots ,t_n \} \in {\mathbb {R}}^{(n\times 8)} \end{aligned}$$

where $\Delta t$ is the sampling interval, one day in this paper.

Regarding ${\mathbb {K}}(n;\varvec{\xi }_j) \in {\mathbb {R}}^{(n\times 8)}$ as a matrix, the $m_{th}$ column, denoted as $\mathbf{{k}}^{(m)}(n;\varvec{\xi }_j)$ ($m=1, \ldots ,8$), corresponds to the time series of a principal Gauss curvature of a pseudo physics quantity. For instance, $\mathbf{{k}}^{(1)}(n;\varvec{\xi }_j)$ the 1st column of ${\mathbb {K}}(n;\varvec{\xi }_j)$, means the time series of $\kappa _1$ of the pseudo released energy up to this time $t_n$ at a reference volume $\varvec{\xi }_j$ whereas the 8th column $\mathbf{{k}}^{(8)}(n;\varvec{\xi }_j)$ means the time series of $\kappa _2$ of the pseudo Laplacian’s first term.

To generate the Fourier transform-based new features, this study performed the fast Fourier transform (FFT). The well-established library *FFTW*^20,21^ is used to carry out FFT of the discrete time series of the Gauss curvature-based features of the pseudo physics quantities, i.e., each column of ${\mathbb {K}}(n;\varvec{\xi }_j)$. The FFT generates the resultant set ${F}_{PSD}$ consisting of the power spectral densities $\textbf{p}_{PSD} \in {\mathbb {R}}^{n}$ and the associated frequencies $\textbf{f} \in {\mathbb {R}}^{n}$. In short, ${\mathbb {K}}(n;\varvec{\xi }_j) \rightarrow {F}(n;\varvec{\xi }_j) := \{ \textbf{p}_{PSD}^{(1)},\textbf{f}^{(1)}, \ldots , \textbf{p}_{PSD}^{(8)},\textbf{f}^{(8)} \}.$ Then, for each column, we can remove the DC component and sort the column vectors in descending order with respect to the magnitude of PSD. The resulting sorted set (${\overline{F}}$) from ${F}(n;\varvec{\xi }_j) \rightarrow {\overline{F}}(n;\varvec{\xi }_j) := \{ \overline{\textbf{p}}_{PSD}^{(1)}, \overline{\textbf{f}}^{(1)},\ldots ,\overline{\textbf{p}}_{PSD}^{(8)},\overline{\textbf{f}}^{(8)} | \; {\overline{p}}^{(m)}_{i-1} \ge {\overline{p}}^{(m)}_{i}, \forall {i}\in [1,n]\}$ where $\overline{\textbf{p}}_{PSD}^{(m)}=({\overline{p}}^{(m)}_{1},\ldots ,{\overline{p}}^{(m)}_{n})^\text {T}$. Thus, ${\overline{p}}^{(m)}_{i}$ is the $i_{th}$ entity of the sorted column vector $\overline{\textbf{p}}_{PSD}^{(m)}$ in descending order. $\overline{\textbf{f}}^{(m)}$ is the sorted frequency vector according to the $\overline{\textbf{p}}_{PSD}^{(m)}$.

To generate practically meaningful features, amongst many peaks in the power spectra, this paper extracted the top 10 amplitudes and the associated frequencies. FT-based new feature set is denoted as ${\mathbb {F}}(n; \varvec{\xi }_i)\in {\mathbb {R}}^{10\times 16}$, ${\overline{F}}(n;\varvec{\xi }_j) \rightarrow {\mathbb {F}}(n; \varvec{\xi }_i):= \{ \overline{\textbf{p}}_{top}^{(1)}, \overline{\textbf{f}}^{(1)}_{top},\ldots ,\overline{\textbf{p}}_{top}^{(8)}, \overline{\textbf{f}}^{(8)}_{top}| \; \overline{\textbf{p}}_{top}^{(m)} \subset \overline{\textbf{p}}_{PSD}^{(m)}, \overline{\textbf{f}}^{(m)}_{top} \subset \overline{\textbf{f}}^{(m)} \}.$

**Figure 2:**
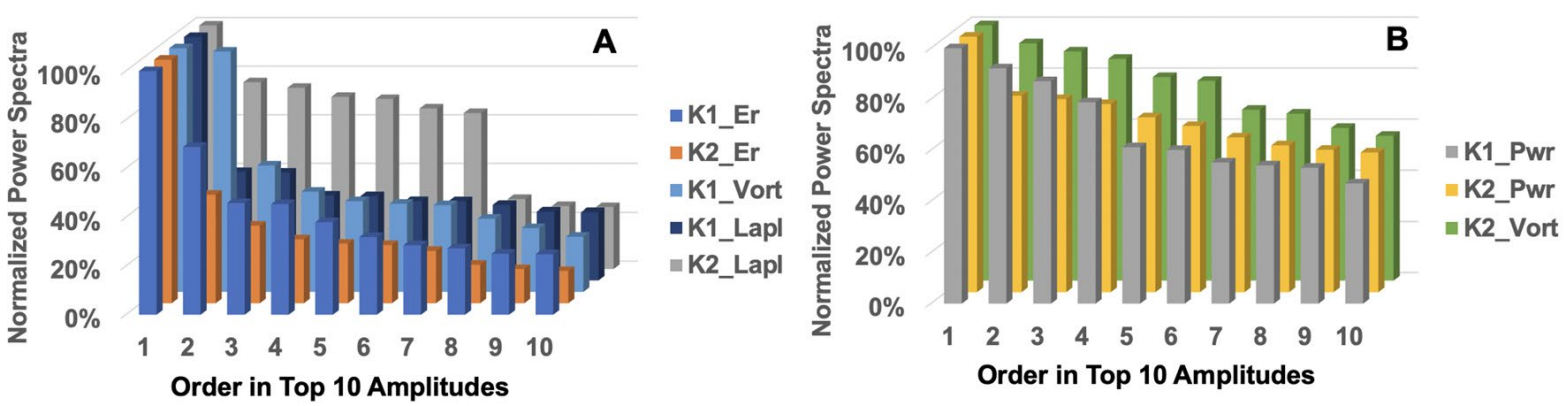
Normalized power spectra of the top 10 amplitudes and their frequencies after sorting the FFT results. (**A**) Rapidly decreasing relative energy levels of top 10 peaks from FFT of Gauss curvatures (denoted as K1 and K2). (**B**) Slowing decreasing relative energy levels. Er: The pseudo released energy, Vort: the pseudo vorticity, Lapl: the pseudo Laplacian, and Pwr: the pseudo power.

There is no strict restriction to how many top amplitudes are selected. This paper adopts up to top 10 since it can encompass sufficient energy of the total energy of the input signal. For instance, Fig. 2A shows that the top 10 peaks sufficiently large energy level and that beyond the ten peaks, the energy level decreases to 20% of that of the largest peak. Figure 2B shows exceptions when the 10th peak’s energy level does not decrease to below 50% of that of the largest peak. The results of this paper support that the inclusion of the top 10 amplitudes and their frequencies in the FT-based features is successful to distinguish and learn hidden rules of the imminent extreme EQs. Including more peaks (thus more energy) will be straightforward, and investigation into their impacts shall be a future research topic.

### Data-driven prediction rules for individual large EQs

This paper pursues the purely data-driven prediction rules that are customized for individual large EQs, being independent of existing magnitude prediction models^22–25^, or earthquake forecasting methods^26–33^. Overall architecture of the adopted hidden rule-learning ML algorithm is illustrated in Fig. 1A(V). The generality of the hidden rule-learning approach shown in Fig. 1 has been demonstrated with complex physics phenomena at diverse scales from nano^34^, to micro^35^, to composite structures^36,37^, and to the Earth lithosphere^17^.

Prediction rules are unraveled by the Glass-Box Rule-Learning algorithm that uses the multi-layered data (Fig. 1A(V)). The role of “Scientist in the Loop” is to monitor the rule-based predictions and help decide whether to recommend appending the identified best-so-far rule into the storage (i.e., global memory for future inheritance and predictions) or not. For instance, some predictions may have good fitness scores numerically, but their prediction plots may not satisfy the domain expert’s knowledge. Then, the “Scientist in the Loop” may queue for additional rule-learning by changing ML-control parameters or expanding search spaces. Since each large EQ has its own prediction rule in the proposed approach, such a re-learning can be done separately and multiple times, specifically for the EQ. This scientist’s role augments the data-driven rule-learning process to better comply with domain science.

In the previous work of the author^17^, the best-so-far prediction rule was identified by a multiplicative combination of cubic regression spline (CRS)-based LFs of (i) the pseudo released energy (its LF is denoted ${\mathcal {L}}_{E}$), (ii) the pseudo power (${\mathcal {L}}_{P}$), (iii) the pseudo vorticity (${\mathcal {L}}_{\omega }$), and (iv) the pseudo Laplacian (${\mathcal {L}}_{L}$). The CRS-based LF can leverage its high flexibility^38,39^, and its general form is given in “Methods”. CRS-based LFs can embrace constant shift, linear, and nonlinear curves^38,39^. Thus, the best-so-far data-driven prediction rule without the Fourier transform-based features (denoted $M_{CRS}^{(t+1)}$) is given by

4 $$\begin{aligned} M_{CRS}^{(t+1)}(\varvec{\xi }_j) = {\mathcal {L}}_{E}(E_r^{*(t)}) {\mathcal {L}}_{P}\left( \text {Sg}\left( e^2 \frac{\partial E_r^{*(t)}}{\partial {t}} \right) \right) {\mathcal {L}}_{\omega }\left( \text {Sg}\left( e^2\omega _{\lambda } \right) \right) {\mathcal {L}}_{L}\left( \text {Sg}\left( 10^{-4} \frac{\partial ^2 E_r^{*(t)}}{\partial {\lambda ^2}} \right) \right) \end{aligned}$$

where $E_r^{*(t)}$ is the best-so-far pseudo released energy at epoch *t* and at the reference volume $\varvec{\xi }_j$. The free parameters associated with the best-so-far CRS LFs ${\mathcal {L}}_{E}, {\mathcal {L}}_{P}, {\mathcal {L}}_{\omega }$, and ${\mathcal {L}}_{L}$ are denoted by $\varvec{\uptheta }_{E}$, $\varvec{\uptheta }_{P}$, $\varvec{\uptheta }_{\omega }$, and $\varvec{\uptheta }_{L}$, respectively. $\text {Sg}(.)$ stands for a typical sigmoid function. The detailed rationales for the data-driven rules are presented in Ref.^17^.

In this study, the Fourier bases are used to sharpen the prediction rule. The top 10 frequencies of the principal Gauss curvatures are used in the Fourier bases. The best-so-far data-driven prediction rules with the Fourier transform-based features in conjunction with CRS LFs is denoted by $M_{FT \& CRS}^{(t+1)}$.

5 $$\begin{aligned} M_{FT \& CRS}^{(t+1)}(\varvec{\xi }_j) =&{\mathcal {L}}_{E}\times ( {\mathcal {L}}_{FT}({\kappa _1}^{(E)}) + {\mathcal {L}}_{FT}({\kappa _2}^{(E)}) ) \nonumber \\&\times {\mathcal {L}}_{P} \times ( {\mathcal {L}}_{FT}({\kappa _1}^{(P)}) + {\mathcal {L}}_{FT}({\kappa _2}^{(P)}) ) \nonumber \\&\times {\mathcal {L}}_{\omega } \times ( {\mathcal {L}}_{FT}({\kappa _1}^{(\omega )}) + {\mathcal {L}}_{FT}({\kappa _2}^{(\omega )}) ) \nonumber \\&\times {\mathcal {L}}_{L}\times ( {\mathcal {L}}_{FT}({\kappa _1}^{(L)}) + {\mathcal {L}}_{FT}({\kappa _2}^{(L)}) ) \end{aligned}$$

where ${\mathcal {L}}_{E}, {\mathcal {L}}_{P}, {\mathcal {L}}_{\omega }$, and ${\mathcal {L}}_{L}$ are from Eq. (4); the Fourier-based link functions are give as

6 $$\begin{aligned} {\mathcal {L}}_{FT}({\kappa _1}^{(E)}) = \sum _{i=1}^{10}(a_i \text {cos}(2\pi {\overline{f}}_i^{(1)} \kappa _1^{(E)}) + b_i \text {sin}(2\pi {\overline{f}}_i^{(1)} \kappa _1^{(E)})) \end{aligned}$$

Similarly, all other Fourier-based LFs are given in Supplemental Material. Here, the sorted frequency ${\overline{f}}_i^{(1)}$ is the $i_\text {th}$ entity of $\overline{\textbf{f}}^{(1)}_{top}$. In particular, the Fourier frequencies of $(\kappa _1, \kappa _2)_E$ are used to augment ${\mathcal {L}}_{E}$ as shown in the first line of Eq. (5). The FT-based LFs appear to offer considerable flexibility to the prior best-so-far prediction rule (Eq. 4) and thus improve accuracy of the prediction rules.

It should be noted that all the LFs are customized for individual large EQs in this paper. Rather than a single set of LFs describing all EQs, each large EQ will have its own best-so-far LFs (i.e., specialized prediction rules). This customized approach will be helpful for a future expansion to a reinforcement learning-based evolution of this framework in which unsupervised ML methods can continue learning and improving the best prediction rules (i.e., LFs) for many different large EQs, without human interventions.

### Feasibility test results

This paper expanded the study region compared to the previous study^17^ to wider reference events ($M_w \ge 6.5$) in the West U.S. region (i.e., longitude and latitude in (− 132.5, − 110) and (30, 52.5) [deg], respectively, and depth (− 5, 20) [km]) within the past 40 years from 1980 through 2019.

**Figure 3:**
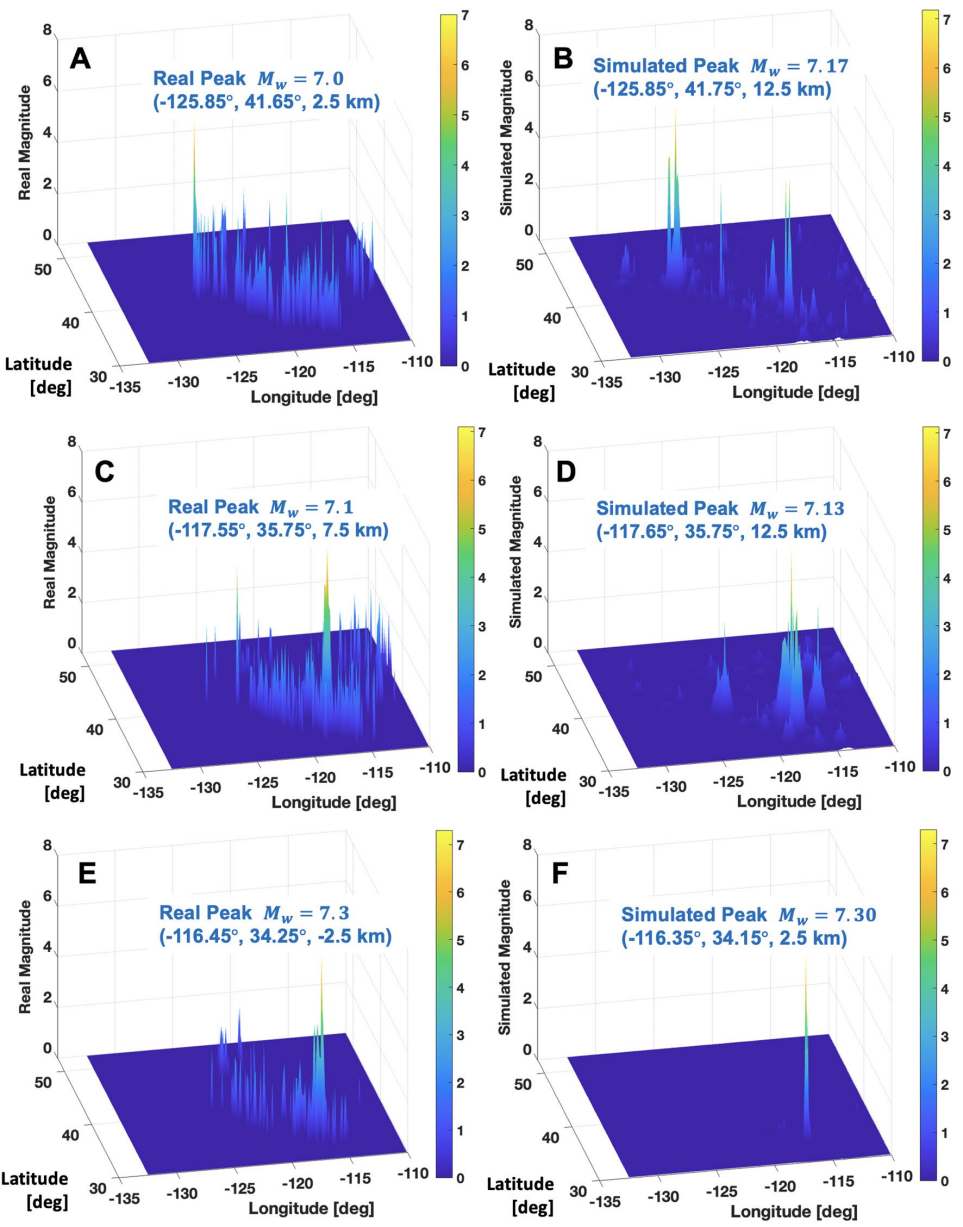
Reproduction of large magnitude events $M_w>7.0$ by using the customized ML-identified data-driven prediction rules with FT-based new features: (**A**,**B**) Observed real and simulated earthquake events on 1991/8/17 (target day ID 1004246); (**C**,**D**) 2019/7/6 (1014431); (**E**,**F**) 1992/6/28 (1004562).

The ML-identified rule uses the observed 10-year data, 30 days before the event without any physics mechanisms or statistical laws. The best-so-far rule appears to be successful in reproducing the next-month earthquake’s location and magnitude 30 days before the event as shown in Fig. 3. The ML-identified rule appears to reproduce the global peak event noticeably well. In addition to Fig. 3, all other ML-driven reproductions of 8 large events of magnitudes ($M_w \ge 7.0$) in the West U.S. region are shown in Figs. S2 and S3. Also, Figs. S4–S6 presents ML-driven reproductions of 9 large events of magnitudes ($M_w \in [6.5, 7.0)$) in the western U.S. region. In some cases, the ML-identified rules reproduce reasonably the global peak’s location and magnitude with a few false small peaks (e.g., Figs. S2A–D). Such false peak reproductions appear more noticeable in events of magnitudes ($M_w \in [6.5, 7.0)$) than events of magnitudes ($M_w \ge 7.0$). For instance, Figs. S5C–F appear to show the wrong reproductions of false peaks. It is related to the limit of the best-so-far ML-identified rules which shall be improved in the future extension. Still, the overall performance is promising since the largest peaks’ locations and magnitudes are reasonably reproduced by the customized data-driven model. Table 2 summarizes the prediction results of individual 8 large earthquakes of magnitude ($M_w \ge 7.0$) and 9 large earthquakes of ($M_w \in [6.5, 7.0)$) using the best-so-far data-driven prediction model. For the 8 large earthquakes of magnitude ($M_w \ge 7.0$), the mean differences in (latitude, longitude, depth, magnitude) between real peak and ML-reproduced peak are ($0.12^{\circ }, 0.15^{\circ }, 4.21 \text { km}, 0.18$). The mean differences increase to ($0.28^{\circ }, 0.51^{\circ }, 5.4 \text { km}, 0.22$) for the 9 large earthquakes of magnitude ($M_w \in [6.5, 7.0)$). These difference underpins the overall accuracy of the best-so-far ML-identified rules in reproducing three-dimensional locations and magnitudes 30 days before the event. But, it also implies that the rule’s accuracy appears to deteriorate for the second largest group of ($M_w \in [6.5, 7.0)$). Uncertainty also increases for this second largest group. For the 8 large earthquakes of magnitude ($M_w \ge 7.0$), the standard deviation of the differences in (latitude, longitude, depth, magnitude) between real peak and ML-reproduced peak are ($0.1^{\circ }, 0.17^{\circ }, 3.19 \text { km}, 0.12$). The standard deviation of differences increase to ($0.22^{\circ }, 0.52^{\circ }, 4.52 \text { km}, 0.15$) for the 9 large earthquakes of magnitude ($M_w \in [6.5, 7.0)$). Improvement of accuracy and underlying uncertainty shall be a natural future extension topic.

The preserved interpretability is noteworthy. The best-so-far prediction rules are remembered by storing all the free parameters of the LFs. By retrieving the parameters and plugging them into the corresponding LFs’ expressions (e.g., Eqs. 6 or 11), one can investigate and interpret individual physical terms and their behavior (e.g., Fig. S8 of the author’s prior work^17^). During the rule-learning process, the poor-performing combinations of features and their LFs are rejected. By doing so, this approach can help improve physical interpretation of the ML-identified rules. In particular, the best-performing prediction rule (Eq. 5) turns out to select the pseudo vorticity’s first term $\omega _{\lambda }$ and the pseudo Laplacian’s first term $\frac{\partial ^2 E_r^{*(t)}}{\partial {\lambda ^2}}$ out of many other feature terms (e.g., $\omega _{\lambda }, \omega _{\phi }, \omega _{h}, \sqrt{\omega _{\lambda }^2 + \omega _{\phi }^2}$, $\frac{\partial ^2 E_r^{*(t)}}{\partial {\phi ^2}}, \frac{\partial ^2 E_r^{*(t)}}{\partial {h^2}}$, $\nabla _g^2 E_r^{(t)}$, and so on). Physically, $\omega _{\lambda }$ may describe the slow rotational motion about the longitudinal axis, and the directions of the western U.S. region’s plate motions and the known major faults are roughly parallel or normal to the longitudinal axis. Therefore, any accurate data-driven prediction rules, if properly unraveled, should be able to highlight certain salient physical terms, and such favorable capabilities appear to be confirmed. Purely based on data, this paper’s best prediction rules pinpoint salient feature terms, underpinning the preservation of physical intepretability.

**Table 2:** Individual large earthquake reproductions using the best-so-far customized data-driven models with FT-based new features. 8 events of $M_w \ge 7.0$ and 9 events of $M_w \in [6.5, 7.0). \Delta M_w$ and $\Delta \varvec{\xi }$ are the absolute differences in magnitude and location between the real observation and the predicted peaks, respectively. Some common names of EQs are included. The coordinates ($\lambda , \phi , h$) of all the observed real peaks in this table are the same as those in the USGS catalog database. All other comparison plots between predicted and observed peaks of this paper present the center coordinates of the reference volume ($\Delta \lambda = \Delta \phi = 0.1^{\circ }; \Delta h = 5$ km) that contains the peak.

Target day ID (date)			$M_w$	$\Delta M_w$	$\lambda (^{\circ })$	$\phi (^{\circ })$	h (km)	$\Delta \xi$(km)
1004246	(1991/8/17)	Observed real	7		− 125.86	41.68	1.3	
		Predicted	7.17	0.17	− 125.85	41.75	12.5	13.7
1004498	(1992/4/25)	Observed real	7.2		− 124.23	40.33	9.86	
	Cape Mendocino	Predicted	6.83	0.37	− 124.35	40.45	12.5	19.0
1004562	(1992/6/28)	Observed real	7.3		− 116.44	34.2	− 0.097	
	Landers	Predicted	7.3	0	− 116.35	34.15	2.5	11.7
1005357	(1994/9/1)	Observed real	7		− 126.3	40.41	4.97	
		Predicted	6.81	0.19	− 126.85	40.25	7.5	63.7
1007228	(1999/10/16)	Observed real	7.1		− 116.25	34.6	13.73	
	Hector Mine	Predicted	6.9	0.2	− 116.25	34.95	7.5	39.3
1009297	(2005/6/15)	Observed real	7.2		− 125.95	41.29	16	
		Predicted	6.97	0.23	− 126.15	41.15	17.5	27.2
1011051	(2010/4/4)	Observed real	7.2		− 115.3	32.29	9.99	
	El Mayor–Cucapah	Predicted	6.99	0.21	− 115.15	32.35	12.5	18.1
1014431	(2019/7/6)	Observed real	7.1		− 117.6	35.77	8	
	Ridgecrest	Predicted	7.13	0.03	− 117.65	35.75	12.5	7.5
1004211	(1991/7/13)	Observed real	6.9		− 125.64	42.18	11	
		Predicted	6.47	0.43	− 126.85	41.75	2.5	142.9
1005130	(1994/1/17)	Observed real	6.7		− 118.54	34.21	18.2	
	Northridge	Predicted	6.63	0.07	− 118.25	34.35	7.5	37.3
1005528	(1995/2/19)	Observed real	6.6		− 125.76	40.59	4.62	
		Predicted	6.45	0.15	− 125.65	40.35	7.5	29.5
1008756	(2003/12/22)	Observed real	6.5		− 121.1	35.7	8.38	
	San Simeon	Predicted	6.54	0.04	− 121.05	35.85	12.5	18.0
1009072	(2004/11/2)	Observed real	6.7		− 128.77	49.28	10	
		Predicted	7.06	0.36	− 127.85	49.45	12.5	104.0
1012487	(2014/3/10)	Observed real	6.8		− 125.13	40.83	16.44	
	Ferndale	Predicted	6.73	0.07	− 125.55	40.85	2.5	48.8
1012532	(2014/4/24)	Observed real	6.5		− 127.73	49.64	10	
		Predicted	6.77	0.27	− 126.35	50.35	7.5	172.4
1013491	(2016/12/8)	Observed real	6.6		− 126.19	40.45	8.45	
		Predicted	6.77	0.17	− 126.35	40.95	7.5	58.3
1014174	(2018/10/22)	Observed real	6.8		− 129.29	49.33	10	
		Predicted	7.23	0.43	− 129.25	49.45	7.5	14.3

**Figure 4:**
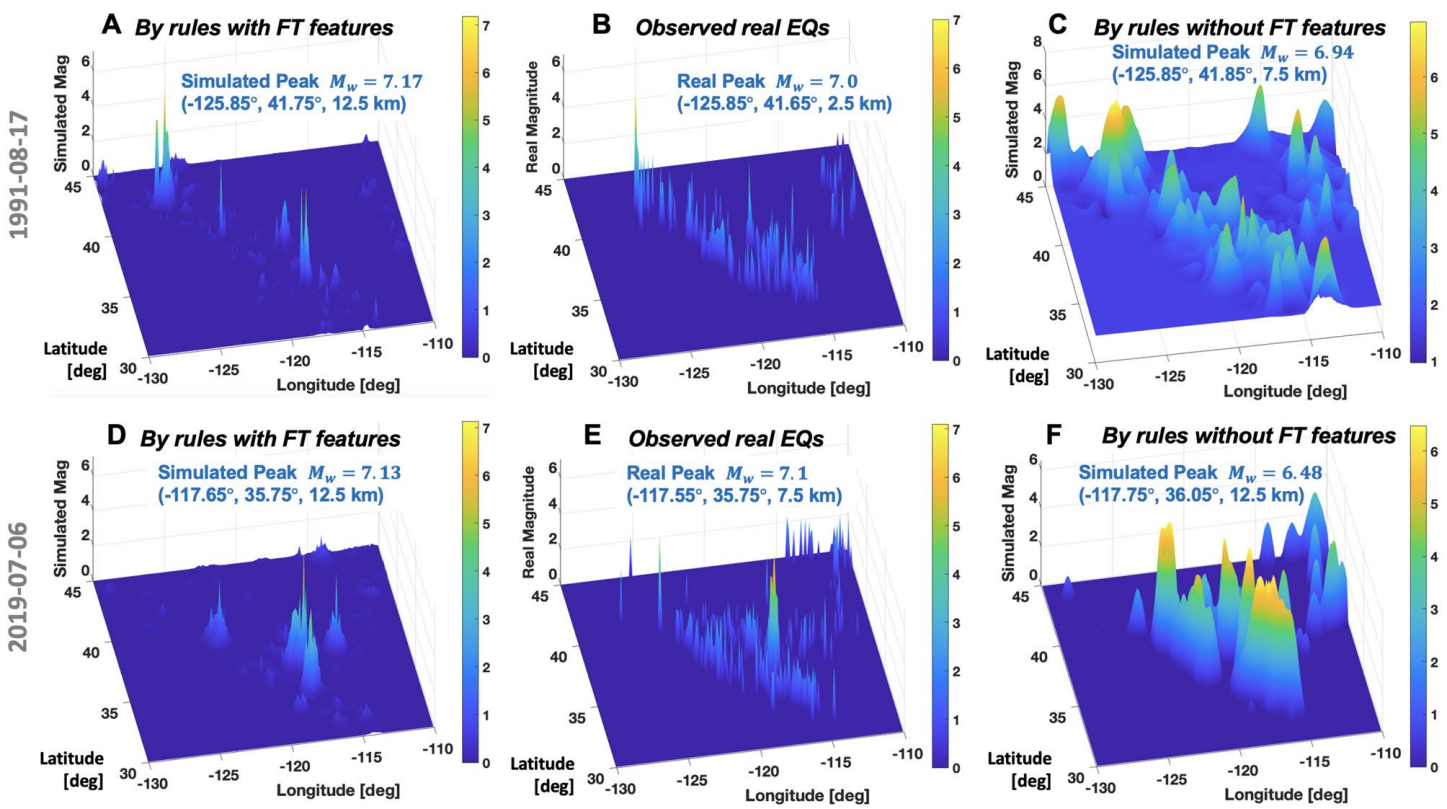
Positive role of FT-based new features in improving prediction accuracy of the best-so-far rules: (**A**–**C**) 1991/8/17 (1004246); (**D**–**F**) 2019/7/6 (Target date ID 1014431). (**A**) and (**D**) harness a combination of Fourier series-based LFs and CRS-based LFs by Eq. (5) whereas (**C**) and (**F**) utilize CRS-bases LFs of the four pseudo physics quantities by Eq. (4).

### Conclusion

The inclusion of FT-based features in the prediction rules appears to be effective to improve accuracy of large EQ’s location and magnitude 30 days before the event. Figure 4 compares the positive impact of the FT-based features. Also, the FT-based features appear to help the ML-identified best-so-far rules to sharpen the predicted magnitude distributions and remove the incorrect peaks. For instance, these positive roles of FT-based features can be clearly seen from comparison between Fig. 4A,C and between Fig. 4D,F.

The improvement can be also confirmed by the sharpened distribution of the absolute errors between real and predicted magnitudes. For instance, Fig. S7 compares the absolute magnitude errors ($|\Delta M_w|$) from predictions without and with FT-based features in the best-so-far rules.

To some extent, it is an anticipated result due to two reasons. FT-based features’ many LFs (${\mathcal {L}}_{FT}$) and the associated free parameters can offer additional fitting power to prediction like a deep learning model with more neurons. Also, many ${\mathcal {L}}_{FT}$’s can be regarded as many higher terms in the Fourier series (here, up to 10 harmonics) which can contribute to smooth fitting strength.

To incorporate the FT-based features, the ML-identified rules should have additional Fourier bases like Eq. (6). When prediction rules use only CRS-based LFs (Eq. 4), the incorrect peaks and over-smoothing issues remain (see Fig. 4C,F). In contrast, the combination of smooth CRS-based LFs ${\mathcal {L}}_{i}, i= (E, P, \omega , L)$ and the Fourier series-based LFs ${\mathcal {L}}_{FT}$ in Eq. (5) can offer enhanced accuracy of reproducing large rare peaks without incorrect peaks and over-smoothing issues (see Fig. 4A,D). The Gibbs phenomenon (i.e., over/undershoot issues near a jump discontinuity) appears not in effect at the present prediction rules using the Fourier series-based LFs. This may be attributed to the fact that the large EQs in this framework are regarded as “point sources” not lines, thus not necessarily leading to sudden discontinuities of target distributions. In the future extensions, when EQs are regarded as 2D line sources, the Gibbs phenomenon may negatively affect the predictions, which shall be addressed later.

Also, in the future investigations, comprehensive validations of the ML-identified prediction rules should be done to confirm general applicability to a wide range of EQ sizes. For instance, Fig. S8 shows preliminary test predictions of “quiet” period without large EQs ($M_w <5.5$ during the period) by using the best-so-far rules. Fig. S8A shows the prediction 33 days before the Ridgecrest EQ (2019/7/6; $M_w=7.1$); Fig. S8B, 32 days before. No false alarms with spurious large EQ predictions are detected. Since the proposed approach trained and unraveled all the prediction rules with large EQs data ($M_w\ge 6.5$), this preliminary test result appears promising. But, to draw a concrete conclusion about the proposed approach and also to be practically meaningful tool (like^28,32^), future extension should conduct comprehensive tests over broad ranges of EQs.

The outcomes of this study add a new dimension to research for predicting individual large EQs. Gauss curvature-based unique signatures of large EQs may be remembered and distinguished by unsupervised ML methods while the data-driven prediction rules can be better customized for individual large EQs with new data. The overall processes can be managed and evolved by another global ML like reinforcement learning, thereby shedding light on purely data- and ML-driven predictions of large EQs. The handshake among ML methods, Fourier, and Gauss may help answer the long-standing enigma of seismogenesis.

## Methods

### Data preparation

This study collected and processed raw earthquake catalog data available in Ref.^18^ from January 1980 through October 2019. Without any prejudice, all the recorded earthquakes within the past 40 years are included, and the total number of earthquakes is 1,895,190. According to the calendar-based date, all earthquakes within one day is stored in one data file. The day-based earthquake catalog data file is named as 1,000,000 for January 1st, 1980, 1,000,001 for January 2nd, 1980, and so on. Each file contains the number of data points in the file followed by longitude, latitude, depth, and magnitude of each earthquake. As illustrated in Fig. S1, one epoch is defined as 30-day time range. All earthquakes within the 30-days window are considered to belong to the same epoch. A frame of epochs consists of many consecutive epochs and serves as the training base for rule-learning glass-box machine learning algorithm. Within one frame of epochs, the last epoch is used as a target while all the previous epochs are used for training of hidden rules. As illustrated in Fig. S1A, the target epoch is completely disjointed from the frame of epochs used for training and rule-learning. As explained in Fig. S1, this study sets one-day interval between consecutive frames of epochs. By marching frames of epochs with one-day increment, this paper can dramatically increase the number of total frames of epochs to 14,600. For interested researchers, all the processed data sets of the refined epochs with one-day interval are publicly available at^40^.

This paper focuses on prediction rule-learning about a large target EQ ($M_w \ge 6.5$) positioned at the last day of the target of epoch (Fig. S1A). Thus, all ML-identified rules of this paper are specifically trained to predict a future large event 30 days before the target EQ (i.e., D-30 case in Fig. S1B). In the future extension, shorter time-window predictions (e.g., a few days ahead) shall be possible by placing the target EQ at the earlier positions of the target epoch (D-2 or D-1 cases in Fig. S1B). In contrast, by defining wider target epochs, longer time-widow predictions (e.g., months or years ahead) shall be also possible, which will be meaningful for complementing the existing long-term EQ forecasting methods.

### Data transformation from raw EQ catalog into spatio-temporal information index

Temporal convolution is carried out after spatial convolution is done as

7 $$\begin{aligned} {\overline{II}}_{ST}^{(t)}(\varvec{\xi }_{j}; L_k, T_l) = \int { \omega (\tau ; T_l) {\overline{II}}_S^{(t_{past})}(\varvec{\xi }_{j}; L_k) dt_{past}} \end{aligned}$$

where the one-dimensional (1D) Gaussian weight $\omega (\tau ; T_l) = (T_l(2\pi )^{1/2})^{-1}\exp \left( -\frac{\tau ^2}{2T_l^2}\right)$; $\tau = |t-t_{past}|, t \ge t_{past}$, meaning the time gap between the current and the past time. And, the spatial convolution is done by

8 $$\begin{aligned} {\overline{II}}_S^{(t)}(\varvec{\xi }_{j}; L_k) = \int _{\text {V}} { \omega (\varvec{\xi }_j, {{\textbf {x}}}_{i}^{(t)}; L_k) M_{i}^{(t)}/10 \; d{{\textbf {x}}}} \end{aligned}$$

where the 3D Gaussian weight $\omega (\varvec{\xi }_j, {{\textbf {x}}}_{i}^{(t)}; L_k) = (L_k(2\pi )^{1/2})^{-N}\exp \left( -\frac{|{{\textbf {x}}}_{i}^{(t)} - \varvec{\xi }_j|^2}{2L_k^2}\right)$; V means the entire lithosphere domain under consideration. Here, $T_l\;(l=1,\ldots ,n_T)$ and $L_k\;(k=1,\ldots ,n_L)$ are influence ranges in time and 3D space, respectively. Therefore, there can be at most ${n_L}\times {n_T}$ spatio-temporal IIs at one reference volume and a time. Following the preliminary investigations done in Ref.^17^, this study adopts $n_L=2$ with $L_1=10$ (km) and $L_2=25$ (km) while $n_T=2$ with $T_1=3$ (month) and $T_2=6$ (month). This combination appears to lead to the best-so-far prediction performance since it embraces dual impacts of close and far EQs both in three-dimensional spatial domain and in one-dimensional temporal domain.

### Flexible and expressive link functions

Pursuing the interpretability, this paper adopts an expressive link function (LF) using transparent, flexible bases that can describe a mathematical expression between input features and the hidden rules as output. LF is denoted as ${\mathcal {L}}$ where $\varvec{\uptheta }$ is a set of free parameters prescribing the LF. The cubic spline regression (CRS) curves consist of a few cubic polynomials connected at knots so that the curves are continuous up to the second derivatives^38^. For practical cubic spline bases^39^ (denoted as $b_i$), LFs look like

9 $$\begin{aligned}{} & {} {\mathcal {L}}({\overline{II}}_{ST};~{{\textbf {a}}},{{\textbf {x}}}^*) = \sum _i^p{a_{i} b_{i}({\overline{II}}_{ST})} \end{aligned}$$

10 $$\begin{aligned}{} & {} {CRS-Based LF: }{\mathcal {L}}^{(k,l)}({\overline{II}}_{ST}^{(t)}(\varvec{\xi }_{j}; L_k, T_l); ~\varvec{\uptheta }^{(k,l)}) = \sum _{i=1}^p{a_{i}^{(k,l)} b_{i}^{(k,l)}({\overline{II}}_{ST}^{(t)}(\varvec{\xi }_{j}; L_k, T_l))}; \end{aligned}$$

where $$b_{1}(x) = 1, b_{2}(x) = x,$$ and

11 $$\begin{aligned} b_{i+2}(x) = \frac{[(x_i^* - \frac{1}{2})^2 - \frac{1}{12}][(x-\frac{1}{2})^2 - \frac{1}{12}]}{4} - \frac{[(|x-x_i^*|-\frac{1}{2})^4 - \frac{1}{2}(|x-x_i^*| - \frac{1}{2})^2 + \frac{7}{240}]}{24}, \end{aligned}$$

for $i=1 \ldots p-2.$ Here, $x_i^*$ is $i_{th}$ knot location. To fully describe one LF, we need to identify $p + (p-2)$ unknowns, i.e. ${{\textbf {a}}} = \{a_1, \ldots ,a_p\}$ and ${{\textbf {x}}}^* = \{x_1^*, \ldots ,x_{(p-2)}^*\}.$ The CRS-based LF can accommodate simple monotonic rule to highly nonlinear rule. Importantly, the adopted CRS is not used for the direct “regression” but for the transparent expression searching.

### Bayesian evolutionary algorithm

This paper adopts the Bayesian evolutionary algorithm to find the total free parameters of hidden rules and also to enable smooth evolution of the rules. In particular, the total free parameters include the parameters of the pseudo released energy rule in Eq. (1), CRS LFs of prediction rules in Eq. (4), and Fourier transform-based LFs of predition rules in Eq. (5). A combination of the fitness-proportionate probability (FPP) scheme of the genetic algorithm and the Bayesian update scheme is at the center of the method (see details in Ref.^17^). On the Bayesian evolutionary framework, in total 71,600 organisms (i.e., candidates for total free parameters of hidden rules) and 20 generations are used, 4 alleles per gene are used, and the variable-wise mutation with rate of 0.005 is used. Preliminary investigations narrow down the best-performing search ranges such that (i) exponential LFs’ two parameters reside in $[0,3]\cup [0,10]$; (ii) CRS LFs’ parameters of five bases all reside in $[-2,2]$ and three knots’ loci are $[0, 1/3]\cup [1/3, 2/3]\cup [2/3, 1]$; (iii) Fourier Transform-based LFs’ parameters of Fourier bases all reside in $[-2,2]$. As done in Ref.^17^, the three-fold error measure (i.e., fitness) is based on differences in magnitude, three-dimensional location, and the wrong peaks’ count (false alarms) between real observations and best-so-far rule-driven predictions. The key steps of the Bayesian evolutionary algorithm are presented in Table S2 of Supplementary Information.

### Computation cost

All computational simulations, training, and predictions of this paper were conducted on NOVA, a high-performance computing cluster of Iowa State University. NOVA cluster consists of compute nodes with two 18-Core Intel Skylake 6140, 1.5 TB of fast NVME local storage, and 192 GB of memory. All nodes and storage are connected via Mellanox EDR (100 Gbps) switch. Given one target EQ, the rule-learning simulation used 144 cores and finished within 12 h, which includes all steps: new feature generations, FFT, and rule-learning with Bayesian evolutionary algorithm. Once the best-so-far rule’s free parameters are identified and stored, one separate prediction using top 10 best-so-far rules costs only 4 min with 16 cores.

### Supplementary Information

Supplementary Information.

## Data Availability

The processed 40-years data sets consisting of the month-based epochs and the refined day-based epochs are shared on a cloud storage^40^. Other supplementary data and parallel programs supporting other findings of this paper will be available upon request to the author.
