# Supplementary material for: Sharpen data-driven prediction rules of individual large earthquakes with aid of Fourier and Gauss
Source: Sci Rep. 2023 Sep 25;13:16009. doi: 10.1038/s41598-023-43181-z (PMC10520116; doi:10.1038/s41598-023-43181-z)
Supplement: Supplementary file 1 — Supplementary Information. [file 41598_2023_43181_MOESM1_ESM.pdf]

Sharpen Data-Driven Prediction Rules of Individual Large Earthquakes with Aid of Fourier and Gauss

In Ho Cho[1]

[1]CCEE Department, Iowa State University, Ames, IA 50011, USA

1 Refined frames of epochs with day-based increment

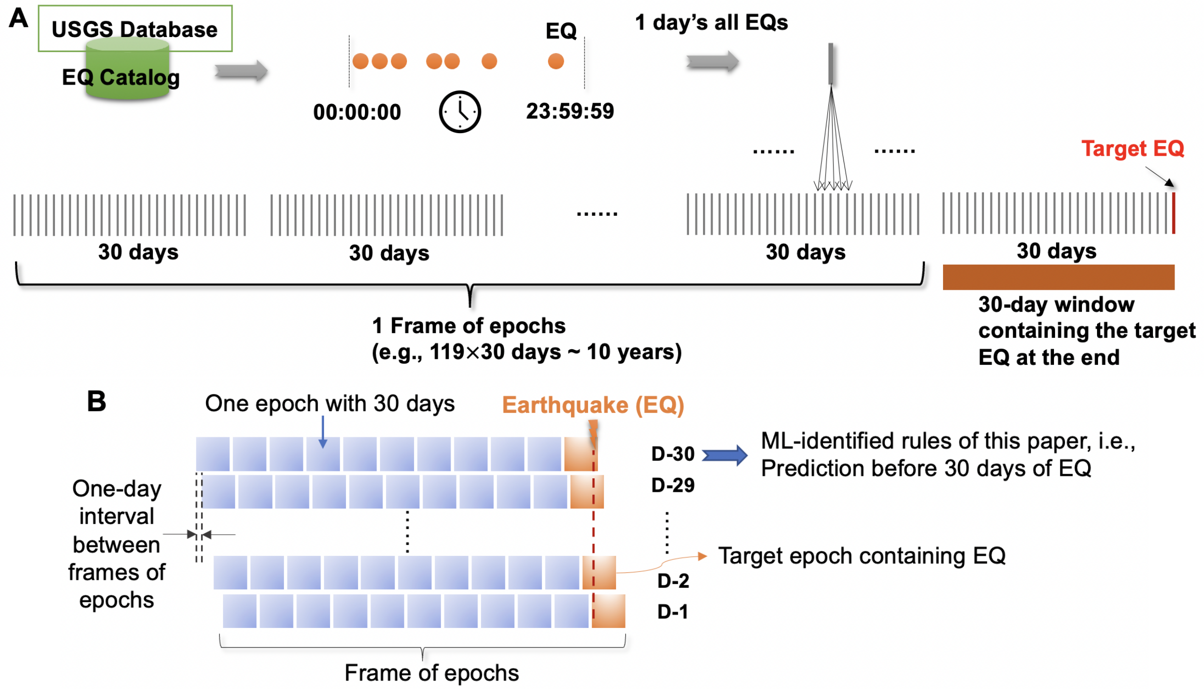

Fig. S1. (A) Detailed procedure of generating a frame of epochs from USGS earthquake catalog database; (B) Refined frames of epochs with one-day interval. 10-years data before the target earthquake (EQ) are used for prediction rule-learning. This paper focuses on prediction of target EQ 30 days before.

2 Summary of equations and formulas of salient steps

Table S1. Key Equations and Formulas

[Data Transformation-I] Spatio-Temporal Information Index

From USGS (*18*), prepare raw data matrix of all past EQs, $\{\lambda, \phi, -h, M\}_i^{(t)}$

• $II_{local} \in \mathbb{R}[0,1]$, $II_{local}{}^{(t)}(\mathbf{x}_i^{(t)}) = M_i^{(t)}/10$ where $\mathbf{x}_i^{(t)} = (x, y, z)_i^{(t)}$

• $\overline{II}_S^{(t)}(\boldsymbol{\xi}_j; L_k) = \int_V \omega(\boldsymbol{\xi}_j, \mathbf{x}_i^{(t)}; L_k) II_{local}^{(t)}(\mathbf{x}_i^{(t)}) d\mathbf{x}$

where $\omega(\boldsymbol{\xi}_j, \mathbf{x}_i^{(t)}; L_k) = (L_k(2\pi)^{1/2})^{-N} \exp\left(-\frac{|\mathbf{x}_i^{(t)} - \boldsymbol{\xi}_j|^2}{2L_k^2}\right) = \mathcal{N}(\mathbf{x}_{(i)}^{(t)}, L_k^2)$

• $\overline{II}_{ST}^{(t)}(\boldsymbol{\xi}_j; L_k, T_l) = \int \omega(\tau; T_l) \overline{II}_S^{(t_{past})}(\boldsymbol{\xi}_j; L_k) dt_{past}$

where $\omega(\tau; T_l) = (T_l(2\pi)^{1/2})^{-1} \exp\left(-\frac{\tau^2}{2T_l^2}\right) = \mathcal{N}(t, T_l^2)$; $\tau = |t - t_{past}|, t \geq t_{past}$

[Data Transformation-II] Pseudo Physics Features $\mathcal{U}$

• Pseudo Energy: $E_r^{*(t)}(\boldsymbol{\xi}_j) = \max\left[\sum_{k=1}^{n_L=2} \sum_{l=1}^{n_T=2} \mathcal{L}^{(k,l)}(\overline{II}_{ST}^{(t)}(\boldsymbol{\xi}_j; L_k, T_l);\ \theta^{(k,l)}), 0.0\right]$

• Pseudo Power: $E_r' = \frac{\partial E_r^{(t)}(\boldsymbol{\xi}_j)}{\partial t}$

• Pseudo Vorticity: $\boldsymbol{\omega} := \nabla_g \times \left(\nabla_g \frac{\partial E_r^{(t)}(\boldsymbol{\xi}_j)}{\partial t}\right)$

$= \left(\frac{\partial}{\partial \phi}\frac{\partial E_r'}{\partial h} - \frac{\partial}{\partial h}\frac{\partial E_r'}{\partial \phi}, \frac{\partial}{\partial h}\frac{\partial E_r'}{\partial \lambda} - \frac{\partial}{\partial \lambda}\frac{\partial E_r'}{\partial h}, \frac{\partial}{\partial \lambda}\frac{\partial E_r'}{\partial \phi} - \frac{\partial}{\partial \phi}\frac{\partial E_r'}{\partial \lambda}\right)$

where $\nabla_g \mathrm{f}(\boldsymbol{\xi}_j) = \mathbf{J}\nabla \mathrm{f}(\boldsymbol{\xi}_j)$ (see full details in (*17*))

• Pseudo Laplacian: $\nabla_g^2 E_r^{(t)}(\boldsymbol{\xi}_j) = \frac{\partial^2 E_r'}{\partial \lambda^2} + \frac{\partial^2 E_r'}{\partial \phi^2} + \frac{\partial^2 E_r'}{\partial h^2}$

[Data Transformation-III] Gauss Curvature-Based Features $\mathbb{K}$

• $\kappa_1 = H + C;\ \kappa_2 = H - C$

where $H = \frac{GL - 2FM + EN}{2(EG - F^2)}$ and $C = \sqrt{A^2 + B^2}$ (see full details in (*17*))

[Data Transformation-IV] Fourier Transform-Based Features $\mathbb{F}$

• Full details are presented in Table 1 of this paper

3 Link functions of Fourier transform-based new features

$$\mathcal{L}_{FT}({\kappa_2}^{(E)}) = \sum_{i=1}^{10}(c_i\text{cos}(2\pi\overline{f}_i^{(2)}\kappa_2^{(E)}) + d_i\text{sin}(2\pi\overline{f}_i^{(2)}\kappa_2^{(E)})) \tag{12}$$

$$\mathcal{L}_{FT}({\kappa_1}^{(P)}) = \sum_{i=1}^{10}(e_i\text{cos}(2\pi\overline{f}_i^{(3)}\kappa_1^{(P)}) + f_i\text{sin}(2\pi\overline{f}_i^{(3)}\kappa_1^{(P)})) \tag{13}$$

$$\mathcal{L}_{FT}({\kappa_2}^{(P)}) = \sum_{i=1}^{10}(g_i\text{cos}(2\pi\overline{f}_i^{(4)}\kappa_2^{(P)}) + h_i\text{sin}(2\pi\overline{f}_i^{(4)}\kappa_2^{(P)})) \tag{14}$$

$$\mathcal{L}_{FT}({\kappa_1}^{(V)}) = \sum_{i=1}^{10}(o_i\text{cos}(2\pi\overline{f}_i^{(5)}\kappa_1^{(V)}) + p_i\text{sin}(2\pi\overline{f}_i^{(5)}\kappa_1^{(V)})) \tag{15}$$

$$\mathcal{L}_{FT}({\kappa_2}^{(V)}) = \sum_{i=1}^{10}(q_i\text{cos}(2\pi\overline{f}_i^{(6)}\kappa_2^{(V)}) + r_i\text{sin}(2\pi\overline{f}_i^{(6)}\kappa_2^{(V)})) \tag{16}$$

$$\mathcal{L}_{FT}({\kappa_1}^{(L)}) = \sum_{i=1}^{10}(s_i\text{cos}(2\pi\overline{f}_i^{(7)}\kappa_1^{(L)}) + t_i\text{sin}(2\pi\overline{f}_i^{(7)}\kappa_1^{(L)})) \tag{17}$$

$$\mathcal{L}_{FT}({\kappa_2}^{(L)}) = \sum_{i=1}^{10}(u_i\text{cos}(2\pi\overline{f}_i^{(8)}\kappa_2^{(L)}) + v_i\text{sin}(2\pi\overline{f}_i^{(8)}\kappa_2^{(L)})) \tag{18}$$

Table S2. Recap of the Bayesian Evolutionary Algorithm

Three-Fold Error Function ($\mathcal{J}$) in terms of Magnitude, Location, and False Alarms

• $\mathcal{J} = (1 - a_{cnt}) \sum_{\tilde{k} \in Top} \omega_{MD}^{(\tilde{k})} E_{MD}^{(\tilde{k})} / n(Top) + a_{cnt} E_{cnt}$

where definitions of terms are summarized below.

Magnitude & distance error $E_{MD}^{(\tilde{k})} := a_M \text{erf}\left(\frac{|M_{obs}^{(t+1)}(\boldsymbol{\xi}_{\tilde{k}}) - M_{pred}^{(t+1)}(\boldsymbol{\xi}_{k^*})|}{M_{obs}^{(t+1)}(\boldsymbol{\xi}_{\tilde{k}})}\right) + (1 - a_M)\text{erf}\left(\frac{||\boldsymbol{\xi}_{\tilde{k}} - \boldsymbol{\xi}_{k^*}||_2}{r_{max}}\right)$

False alarm error $E_{cnt} := \frac{1}{2}\text{erf}\left(\frac{|n(Top) - n(Top_{pred})|}{n(Top)}\right) + \frac{1}{2}\text{erf}\left(\sum_{\forall \tilde{k} \in Top_{pred}^{-1}} \frac{|M_{thr} - M_{pred}^{(t+1)}(\boldsymbol{\xi}_{\tilde{k}})|/M_{thr}}{n(Top_{pred}^{-1})}\right)$

$Top :=$ a set of indices of reference volumes that contains the sorted real magnitudes $> M_{thr}$

$Top_{pred} :=$ a set of indices of reference volumes of sorted predicted magnitudes $> M_{thr}$

Top_{pred}^{-1} is the complement of the set Top_{pred}

$k^*(\tilde{k}) \in Top_{pred}$ is the closest reference volume to $\tilde{k} \in Top$; $k^*(\tilde{k}) := \text{argmin}_{\forall k \in Top_{pred}} ||\boldsymbol{\xi}_{\tilde{k}} - \boldsymbol{\xi}_k||_2$

$\text{erf}(z) := \frac{2}{\sqrt{\pi}} \int_0^z e^{-t^2} dt$ where $z \in \mathbb{C}$ and $\text{erf}(z) \in [-1, 1]$

Magnitude-dependent scale-up factor $\omega_{MD}^{(\tilde{k})} := \exp(M_{obs}^{(t+1)}(\boldsymbol{\xi}_{\tilde{k}})/10.0)$; $\tilde{k} \in Top$

Other coefficients and hyper-parameters: $a_M = 0.5, a_{cnt} = 0.1, r_{max} = 200$ km,

$M_{thr} = 6.8$ for $M_w \geq 7.0$ group and $M_{thr} = 6.3$ for $M_w \in [6.5, 7.0)$ group

(for full explanations and details see (*17*))

Rule-Learning by Bayesian Evolutionary Algorithm

Learning $M_{FT\&CRS}^{(t+1)}(\boldsymbol{\xi}_j)$ in Eq. (5) means to learn all free parameters Θ of all LFs, $\Theta = (\theta_E, \theta_P, \theta_\omega, \theta_L, \theta_{FT})$ via repetition of solution space exploration, random mutation, fitness-proportionate spawning, and Bayesian inheritance.

• Fitness: $\mathcal{F}(s) = \frac{(1+\mathcal{J}(s))^{-1}}{\sum_{\forall s \in S}[(1+\mathcal{J}(s))^{-1}]}$

where s denotes an individual in the entire generation S

• Bayesian Fitness Score: $\mathcal{F}_B(s) = \frac{1}{\kappa} \frac{\mathcal{F}(s;S^*)\mathcal{F}^*(s)}{\sum_{\forall s \in S^*} \mathcal{F}^*(s)}$

where $\kappa = \sum_{\forall s \in S^*} \frac{\mathcal{F}(s;S^*)\mathcal{F}^*(s)}{\sum_{\forall s \in S^*} \mathcal{F}^*(s)}$

• Probability for selection: $\text{Prob}(\text{parent}_k|s) \propto \mathcal{F}_B(s),\ (k = 1, 2)$

4 Summary of Fourier transform-based new feature generation

As explained in main text, the coordinate vector K consists of the principal Gauss curvatures (κ_1, κ_2) of four pseudo physics quantities at time t at a reference volume $\boldsymbol{\xi}$ as

$$K(t; \boldsymbol{\xi}_j) := ((\kappa_1, \kappa_2)_E, (\kappa_1, \kappa_2)_P, (\kappa_1, \kappa_2)_V, (\kappa_1, \kappa_2)_L)) \tag{19}$$

where E, P, V and L stand for the pseudo released energy, the pseudo power, the pseudo vorticity's first term, and the pseudo Laplacian's first term, respectively, all calculated at time t and a reference volume $\boldsymbol{\xi}_j$. The set of Gauss curvature-based coordinates at $\boldsymbol{\xi}_j$ up to the present time $t_n = n \times \Delta t$ is given by

$$\mathbb{K}(n; \boldsymbol{\xi}_j) := \{K(t; \xi_j) \in \mathbb{R}^8 |\ t = 1, ..., t_n\} \in \mathbb{R}^{(n \times 8)} \tag{20}$$

where Δt is the sampling interval, one day in this paper. Regarding $\mathbb{K}(n; \boldsymbol{\xi}_j) \in \mathbb{R}^{(n \times 8)}$ as a matrix, the m_{th} column, denoted as $\mathbf{k}^{(m)}(n; \boldsymbol{\xi}_j)$ ($m = 1, ..., 8$), corresponds to the time series of a principal Gauss curvature of a pseudo physics quantity. For instance, $\mathbf{k}^{(1)}(n; \boldsymbol{\xi}_j)$ the 1st column of $\mathbb{K}(n; \boldsymbol{\xi}_j)$, means the time series of κ_1 of the pseudo released energy up to this time t_n at a reference volume $\boldsymbol{\xi}_j$ whereas the 8th column $\mathbf{k}^{(8)}(n; \boldsymbol{\xi}_j)$ means the time series of κ_2 of the pseudo Laplacian's first term.

To generate the Fourier transform-based new features, this study performed the column-wise fast Fourier transform (FFT), i.e., each column of $\mathbb{K}(n; \boldsymbol{\xi}_j)$. The FFT generates the resultant set F_{PSD} consisting of the power spectral densities $\mathbf{p}_{PSD} \in \mathbb{R}^n$ and the associated frequencies $\mathbf{f} \in \mathbb{R}^n$. In short,

$$\mathbb{K}(n; \boldsymbol{\xi}_j) \xrightarrow[column-wise]{FFT} F(n; \boldsymbol{\xi}_j) := \{\mathbf{p}^{(1)}_{PSD}, \mathbf{f}^{(1)}, ..., \mathbf{p}^{(8)}_{PSD}, \mathbf{f}^{(8)}\}. \tag{21}$$

Then, for each column, we can remove the DC component and sort the column vectors in descending order with respect to the magnitude of PSD. The resulting sorted set is denoted as

$\overline{F}$ given by

$$F(n;\boldsymbol{\xi}_j) \xrightarrow[by\,PSD]{Sort} \overline{F}(n;\boldsymbol{\xi}_j) := \{\overline{\mathbf{p}}^{(1)}_{PSD}, \overline{\mathbf{f}}^{(1)}, ..., \overline{\mathbf{p}}^{(8)}_{PSD}, \overline{\mathbf{f}}^{(8)} |\, \overline{p}^{(m)}_{i-1} \geq \overline{p}^{(m)}_{i}, \forall i \in [1,n]\} \tag{22}$$

where $\overline{\mathbf{p}}^{(m)}_{PSD} = (\overline{p}^{(m)}_1, ..., \overline{p}^{(m)}_n)^{\mathrm{T}}$. Thus, $\overline{p}^{(m)}_i$ is the i_{th} entity of the sorted column vector $\overline{\mathbf{p}}^{(m)}_{PSD}$ in descending order. $\overline{\mathbf{f}}^{(m)}$ is the sorted frequency vector according to the $\overline{\mathbf{p}}^{(m)}_{PSD}$. In general, $\overline{\mathbf{f}}^{(m)} \neq \overline{\mathbf{f}}^{(m')}$ and $\overline{\mathbf{f}}^{(m)} \neq \mathbf{f}^{(m)}$ for all $m \neq m'$ since the sorting took place column-wise with respect to individual column's PSD.

To generate practically meaningful features, amongst many peaks in the power spectra, this paper extracted the top 10 magnitudes and the associated frequencies. FT-based new feature set is denoted as $\mathbb{F}(n;\boldsymbol{\xi}_i) \in \mathbb{R}^{10\times 16}$,

$$\overline{F}(n;\boldsymbol{\xi}_j) \xrightarrow[Top10]{Extract} \mathbb{F}(n;\boldsymbol{\xi}_i) := \{\overline{\mathbf{p}}^{(1)}_{top}, \overline{\mathbf{f}}^{(1)}_{top}, ..., \overline{\mathbf{p}}^{(8)}_{top}, \overline{\mathbf{f}}^{(8)}_{top} |\, \overline{\mathbf{p}}^{(m)}_{top} \subset \overline{\mathbf{p}}^{(m)}_{PSD}, \overline{\mathbf{f}}^{(m)}_{top} \subset \overline{\mathbf{f}}^{(m)}\} \tag{23}$$

In particular, $\overline{\mathbf{f}}^{(1)}_{top}$ and $\overline{\mathbf{f}}^{(2)}_{top}$ corresponds to the pseudo released energy; $\overline{\mathbf{f}}^{(3)}_{top}$ and $\overline{\mathbf{f}}^{(4)}_{top}$, to the pseudo power; $\overline{\mathbf{f}}^{(5)}_{top}$ and $\overline{\mathbf{f}}^{(6)}_{top}$, to the pseudo vorticity; $\overline{\mathbf{f}}^{(7)}_{top}$ and $\overline{\mathbf{f}}^{(8)}_{top}$, to the pseudo Laplacian.

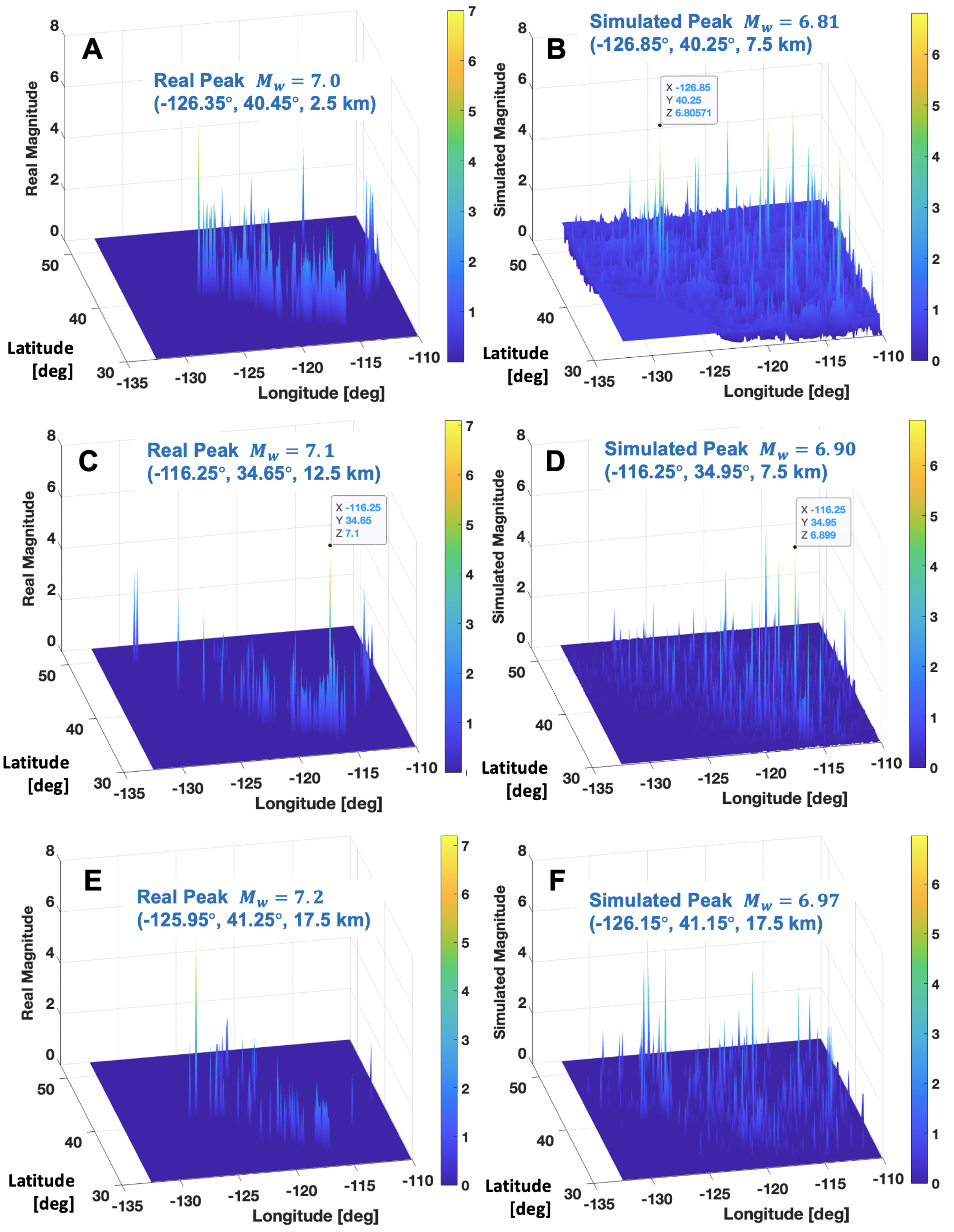

Fig. S2. Reproduction of large magnitude events $M_w > 7.0$ by using the customized ML-identified data-driven prediction rules with FT-based new features: **(A-B)** Observed real and simulated earthquake events on 1994/9/1 (target day ID 1005357); **(C-D)** 1999/10/16 (1007228); **(E-F)** 2005/6/15 (1009297).

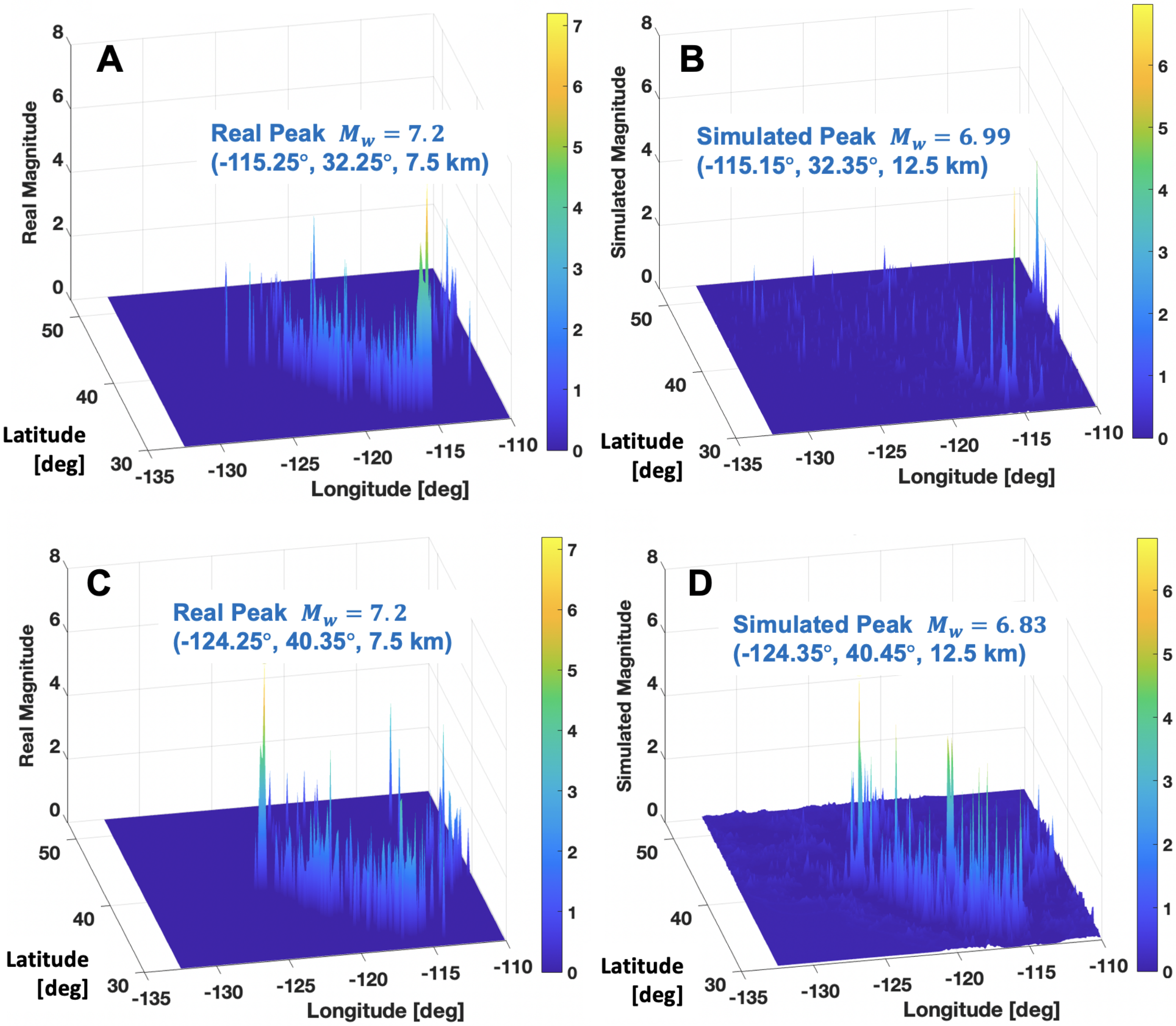

Fig. S3. Reproduction of large magnitude events $M_w > 7.0$ by using the customized ML-identified data-driven prediction rules with FT-based new features: (A-B) Observed real and simulated earthquake events on 2010/4/4 (target day ID 1011051); **(C-D)** 1992/4/25 (1004498).

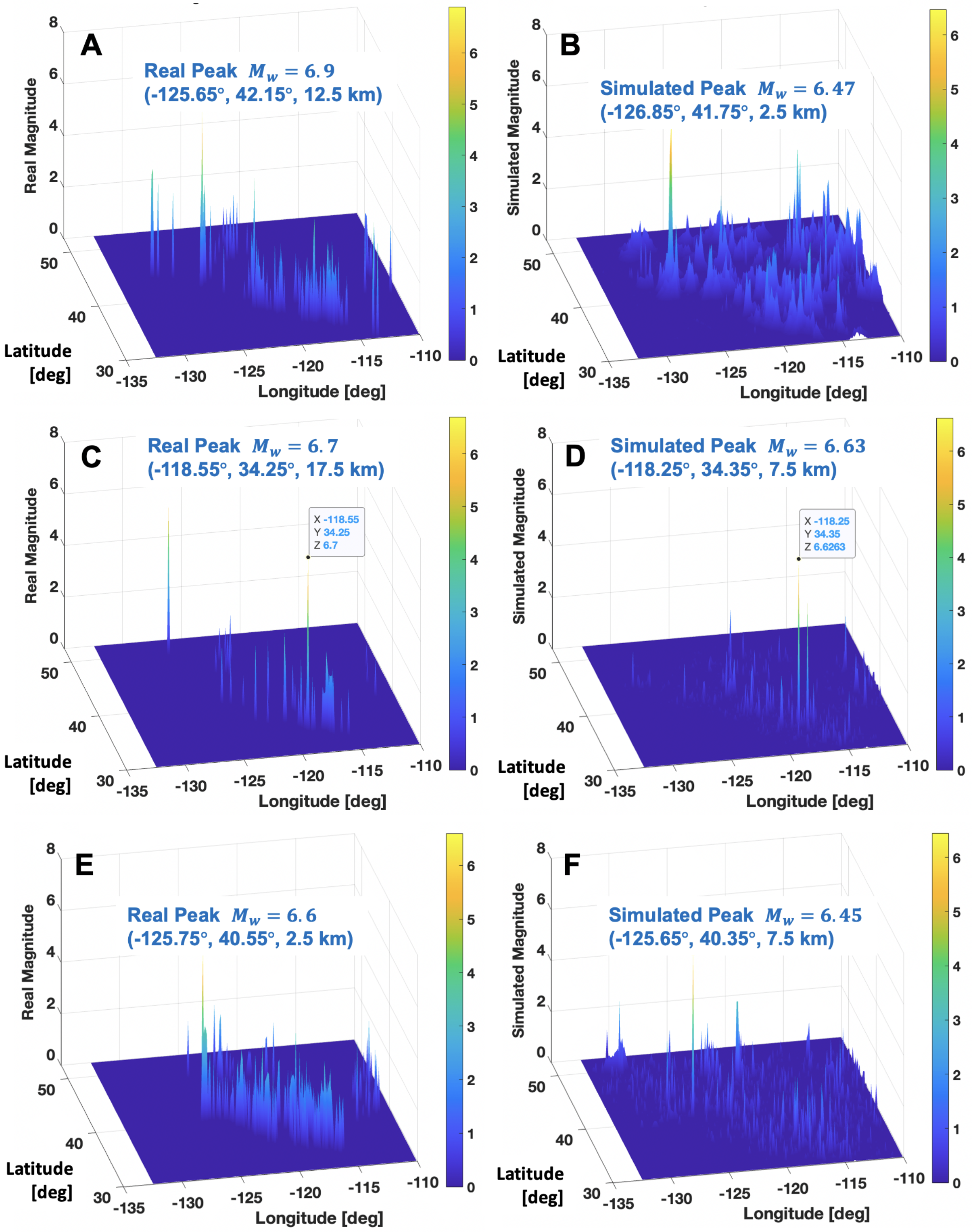

Fig. S4. Reproduction of large magnitude events $M_w \in [6.5, 7.0)$ **by using the customized ML-identified data-driven prediction rules with FT-based new features: (A-B)** Observed real and simulated earthquake events on 1994/7/13 (target day ID 1004211); **(C-D)** 1994/1/17 (1005130); **(E-F)** 1995/2/19 (1005528).

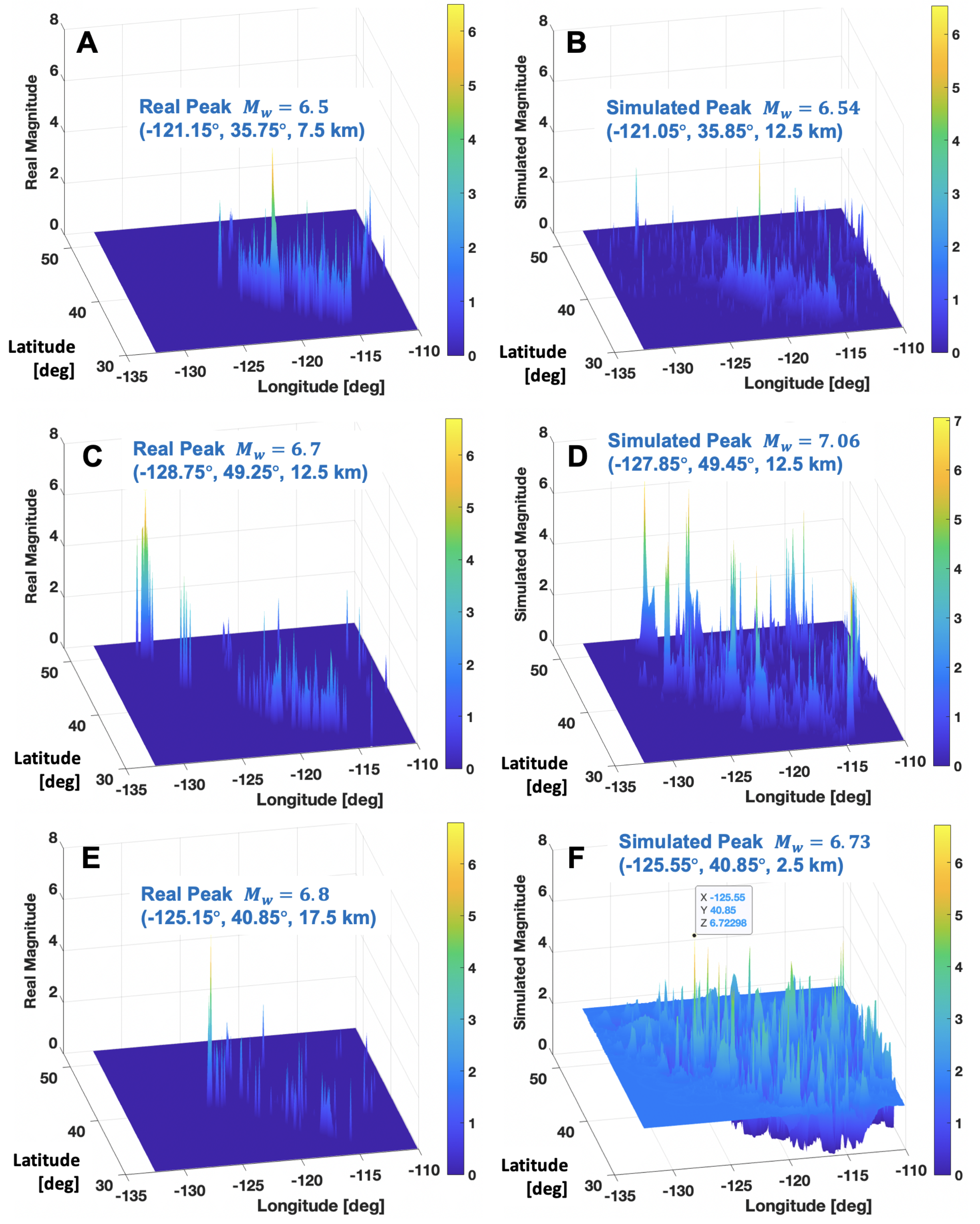

Fig. S5. Reproduction of large magnitude events $M_w \in [6.5, 7.0)$ by using the customized ML-identified data-driven prediction rules with FT-based new features: (A-B) Observed real and simulated earthquake events on 2003/12/22 (target day ID 1008756); **(C-D)** 2004/11/2 (1009072); **(E-F)** 2014/3/10 (1012487).

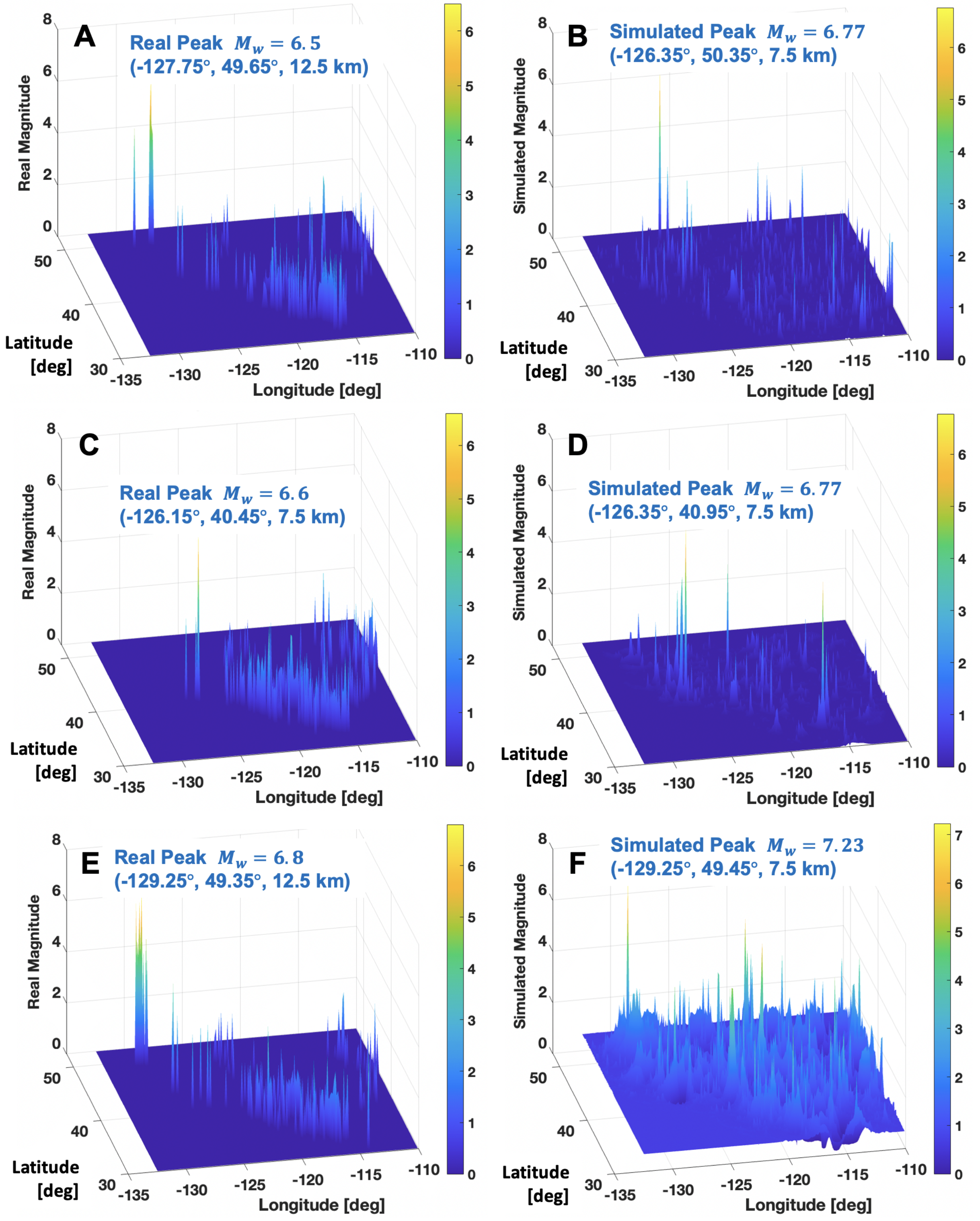

Fig. S6. Reproduction of large magnitude events $M_w \in [6.5, 7.0)$ by using the customized ML-identified data-driven prediction rules with FT-based new features: (A-B) Observed real and simulated earthquake events on 2014/4/24 (target day ID 1012532); **(C-D)** 2016/12/8 (1013491); **(E-F)** 2018/10/22 (1014174).

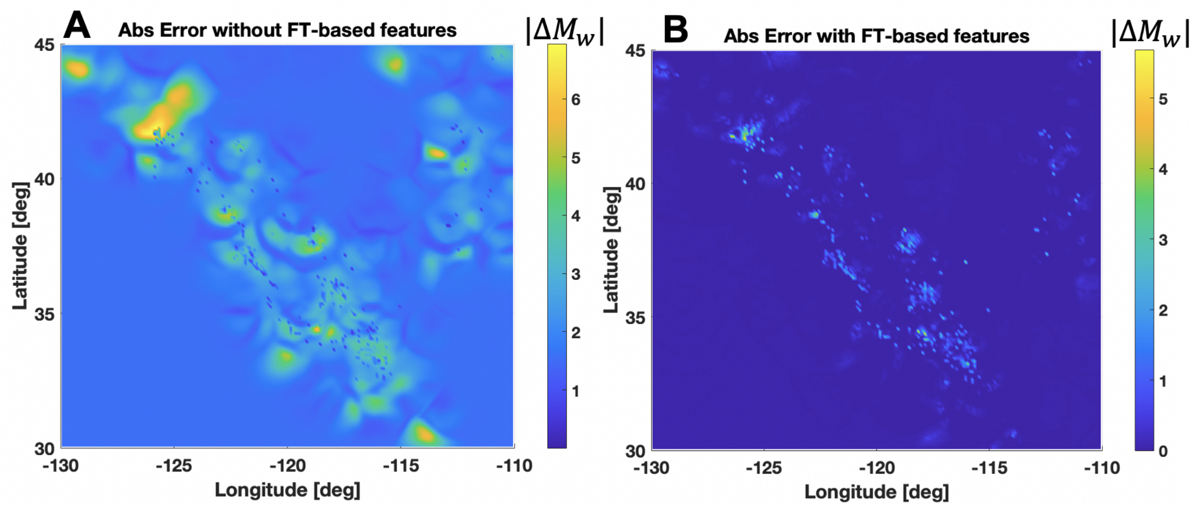

Fig. S7. Absolute errors between real and predicted magnitudes of 1991/8/17 EQ (target day ID 1004246): **(A)** By using the best-so-far rules without FT-based features; **(B)** By the rules with FT-based features.

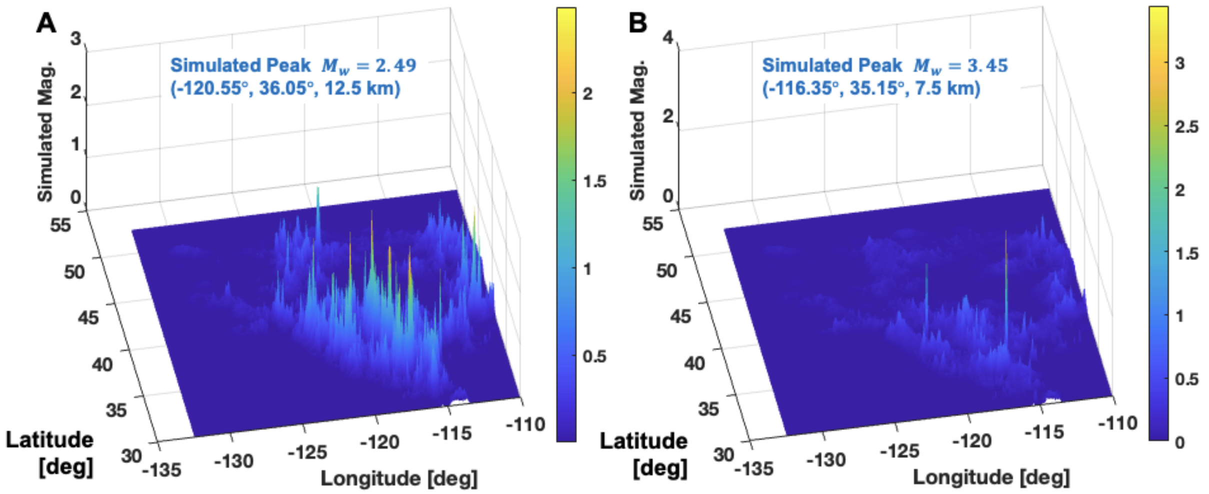

Fig. S8. Preliminary test predictions of small EQs ($M_w < 5.5$) **period by using the best-so-far rules:** **(A)** Predictions 33 days before and **(B)** 32 days before the Ridgecrest EQ (2019/7/6; $M_w = 7.1$). No false alarms with spurious large EQ predictions are detected, but a concrete conclusion requires comprehensive test over all size EQs in the future extension.
